# Potential Utility of Electrical Impedance Myography in Evaluating Age-Related Skeletal Muscle Function Deficits

**DOI:** 10.3389/fphys.2021.666964

**Published:** 2021-05-07

**Authors:** Brian C. Clark, Seward Rutkove, Elmer C. Lupton, Carlos J. Padilla, W. David Arnold

**Affiliations:** ^1^Ohio Musculoskeletal and Neurological Institute (OMNI), Ohio University, Athens, OH, United States; ^2^Department of Biomedical Sciences, Ohio University, Athens, OH, United States; ^3^Division of Geriatric Medicine, Ohio University, Athens, OH, United States; ^4^Department of Neurology, Harvard Medical School, Beth Israel Deaconess Medical Center, Boston, MA, United States; ^5^Myolex Inc., Brookline, MA, United States; ^6^Department of Neurology, Ohio State University, Columbus, OH, United States

**Keywords:** sarcopenia, aging, skeletal muscle, dynapenia, diagnosis

## Abstract

Skeletal muscle function deficits associated with advancing age are due to several physiological and morphological changes including loss of muscle size and quality (conceptualized as a reduction in the intrinsic force-generating capacity of a muscle when adjusted for muscle size). Several factors can contribute to loss of muscle quality, including denervation, excitation-contraction uncoupling, increased fibrosis, and myosteatosis (excessive levels of inter- and intramuscular adipose tissue and intramyocellular lipids). These factors also adversely affect metabolic function. There is a major unmet need for tools to rapidly and easily assess muscle mass and quality in clinical settings with minimal patient and provider burden. Herein, we discuss the potential for electrical impedance myography (EIM) as a tool to evaluate muscle mass and quality in older adults. EIM applies weak, non-detectible (e.g., 400 μA), mutifrequency (e.g., 1 kHz–1 MHz) electrical currents to a muscle (or muscle group) through two excitation electrodes, and resulting voltages are measured *via* two sense electrodes. Measurements are fast (~5 s/muscle), simple to perform, and unaffected by factors such as hydration that may affect other simple measures of muscle status. After nearly 2 decades of study, EIM has been shown to reflect muscle health status, including the presence of atrophy, fibrosis, and fatty infiltration, in a variety of conditions (e.g., developmental growth and maturation, conditioning/deconditioning, and obesity) and neuromuscular diseases states [e.g., amyotrophic lateral sclerosis (ALS) and muscular dystrophies]. In this article, we describe prior work and current evidence of EIM’s potential utility as a measure of muscle health in aging and geriatric medicine.

## Introduction

By 2050, the world’s population aged 60 years and older (the World Health Organization’s cut-off point for an older adult) is expected to total 2 billion (22% of the total population), up from 900 million (12% of the total population) in 2015 (U.N., 2017). Forty-two percent of older adults have one or more physical limitations in performing daily tasks that are essential for independence (Seeman et al., 2010). Age-related muscle wasting, weakness, and diminished muscle quality are important contributors to these physical impairments (Rantanen et al., 1998, 1999, 2000, 2002; Rantanen, 2003; Syddall et al., 2003; Visser et al., 2005; Sasaki et al., 2007; Leong et al., 2015; Duchowny, 2019; McGrath et al., 2019a,b; Jyvakorpi et al., 2020; Warzecha et al., 2020). This age-related degeneration of the muscular (or more precisely neuromuscular) system is commonly referred to as “sarcopenia” or, more conceptually, pathological age-related skeletal muscle function deficits (Cruz-Jentoft et al., 2010, 2019; Correa-de-Araujo et al., 2017, 2020). While voluntary muscle strength can be relatively easily assessed (e.g., handgrip dynamometry), it is heavily influenced by the integrity of the nervous system and, as such, does not necessarily reflect muscle status or health *per se* (Carson, 2018). It is far more challenging to quantify muscle mass and even more so muscle compositional quality [e.g., myosteatosis (fat infiltration), fibrosis] and contractile quality (e.g., intrinsic force-generating capacity normalized to muscle mass, which is also referred to as muscle quality or specific force). Quantitative measures of muscle mass, compositional quality, and contractile quality that can be readily and rapidly applied in the clinic are lacking. Accordingly, there have been recent calls for development and validation of clinically feasible assessment tools to reliably evaluate muscle mass and indices of quality in various settings (Correa-de-Araujo et al., 2017; Buckinx et al., 2018).

Imaging modalities, including MRI, computed tomography (CT), dual-energy x-ray absorptiometry (DXA), and quantitative ultrasonography can be used to obtain insight into muscle size and composition (Clark et al., 2006, 2007; Harris-Love et al., 2014; Erlandson et al., 2016; Correa-de-Araujo et al., 2017). Arguably, imaging by MRI or CT is the current gold-standard approach for assessing muscle mass (Engstrom et al., 1991; Morse et al., 2007; Clark et al., 2018). Similarly, MRI and CT can be used to assess fat infiltration of muscle as an index of muscle compositional quality (Rozenberg et al., 2017; Engelke et al., 2018; Correa-de-Araujo et al., 2020). While MRI and CT are highly accurate and repeatable, they are expensive, housed in a separate facility, and require extensive post-processing that dramatically limits their clinical utility. These challenges severely limit access, real-time screening, and decision-making in the clinic.

In contrast to MRI and CT, DXA, quantitative ultrasound as well as bioelectrical impedance analysis (BIA), are more clinically practical techniques for estimating muscle mass and composition. For instance, DXA-derived estimates of lean body mass strongly correlate with MRI (*r* = 0.88) and CT (*r* = 0.77–0.95) measures of skeletal muscle volume (Kim et al., 2002; Maden-Wilkinson et al., 2013; Heymsfield et al., 2014). However, there is evidence that repeated scans increase measurement error (Hind and Oldroyd, 2013; Knapp et al., 2015) and that DXA-derived measures of change in mass over time correlate poorly with MRI- or CT-derived changes in mass/volume (Nelson et al., 1996; Hansen et al., 2007; Delmonico et al., 2008; Lee and Kuk, 2013; Tavoian et al., 2019). Moreover, DXA instrumentation is still relatively expensive and requires significant physical space, making it less likely to be adopted for in-office, widespread testing. BIA, on the other hand, has many attractive practical features and has the potential to be a point-of-care test (e.g., BIA is very inexpensive, portable, does not require highly trained or experienced personnel; Buckinx et al., 2018). Unfortunately, BIA also has major limitations that have precluded its adoption, which are discussed in more detail below (Buckinx et al., 2018). Quantitative ultrasound can provide indices of muscle size (e.g., thickness and cross-sectional area; Nijholt et al., 2017), as well as proxy estimates of muscle compositional quality (e.g., echogenicity; Reimers et al., 1993; Sipila and Suominen, 1993; Harris-Love et al., 2014). For instance, a recent systematic review concluded that ultrasound is a reliable and valid tool for the assessment of muscle size in older adults (Nijholt et al., 2017). Moreover, echogenicity was recently reported to be associated with intermuscular adipose tissue estimates obtained *via* CT imaging in older men (*r* = 0.73) as well as with measures of muscle strength (*r*-values of ~ 0.4–0.5; Harris-Love et al., 2018). Findings of this nature are particularly notable since ultrasound equipment is often available. However, quantitative ultrasound has limitations that to date appear to have curbed its adoption (e.g., dependence on psychomotor skills of the examiner; Bazzocchi et al., 2016; Correa-de-Araujo et al., 2017).

There are several other technologies/approaches that can provide insight into compositional and contractile muscle quality. For instance, muscle biopsy can yield direct, detailed, and insightful information related to muscle composition. However, muscle biopsy cannot be applied widely for muscle condition assessment due to its invasive nature and the specialized analyses required, and is subject to sampling error since only a very small piece of muscle tissue is obtained. As a measure of muscle contractile quality, electrically stimulated muscle force recordings can provide data related to contractile function (Clark et al., 2006). While useful for research applications, however, electrically stimulated evaluations of contractile function have not been widely adopted clinically for a number of reasons (e.g., it is painful and may not be tolerated by some individuals in routine use) despite the technique being readily available for more than a half-century.

Accordingly, there is a well-recognized unmet medical need for technologies that permit rapid assessment of muscle mass and indices of muscle quality in clinical settings with minimal patient and provider burden (Correa-de-Araujo et al., 2017). Here, we discuss the potential for electrical impedance myography (EIM) outcomes, such as resistance, reactance, and phase angle as a function of input current frequency, to assess age-related skeletal muscle function deficits. EIM has been extensively researched for nearly 2 decades, and has been shown to reflect muscle condition, including presence of atrophy and fatty infiltration (Li et al., 2015; Rutkove and Sanchez, 2019) and is sensitive to simple deconditioning (Tarulli et al., 2009; Rutkove et al., 2017; Roy et al., 2020). Although EIM is not widely discussed in the aging and sarcopenia fields, there is a strong conceptual framework underlying its potential utility for assessing age-related skeletal muscle deficits.

## Basic Measurement Principles of Bioimpedance Technologies

Since the mid-to-late 20th century, specialized electrical impedance methods have found value in a variety of medical and scientific applications, beyond BIA discussed above. For instance, electrical impedance has well-established utility for assessing cardiac output (Lorne et al., 2014), neonatal lung monitoring (Brown, 2003), tumor imaging (Toso et al., 2000; Phillips, 2013), and assessing skin lesions (Aberg et al., 2003). Electrical impedance methods can also be applied for more basic scientific purposes, such as determining the actual electrical properties of tissues and fluids (Gabriel et al., 1996a,b,c).

Body tissues (i.e., muscle) impact the flow of electrical current. Tissue properties can be determined by applying an alternating current to muscle tissue and measuring electrical properties of the generated voltage, namely amplitude and time lag with respect to the applied current. The body tissues actually become components in the electrical circuit and change in current amplitude is associated with resistive behavior of the tissue, that is, the passive compositional structures including free water, connective tissue, and fat (Rutkove and Sanchez, 2019). The time lag of the current is related to capacitive/reactive components, mainly the myofiber membranes (at the frequencies of current typically used), which act as distributed capacitors (Rutkove and Sanchez, 2019). Thus, measuring the electrical properties of voltage (resistance, reactance, and phase angle) relative to the applied current across a broad range of frequencies can yield rich information about muscle structure and integrity. For a full discussion on the electrical engineering principals and mathematics underlying EIM, please see the recent review from Rutkove and Sanchez (2019).

Impedance values are influenced by other factors beyond the intrinsic electrical properties of skeletal muscle being assessed (e.g., conductivity^1^ and relative permittivity^2^). These factors include electrode characteristics and arrangement (e.g., size, material, distance, and shape) and conductor volume (i.e., volume of the limb and position). As such, it is critical to collect EIM data in a standardized fashion. Moreover, while challenging, it is also possible to assess the degree of anisotropy of the tissue. Skeletal muscle tissue is a highly anisotropic due its highly organized cellular and fascicular structure, which results in current flowing more easily along the fibers than across them (Epstein and Foster, 1983). Assuming direct electrode-to-muscle contact, an anisotropy ratio can be calculated (Chin et al., 2008; Kwon et al., 2017), whereby a value of 1.0 indicates a completely isotropic material, and skeletal muscle with a greater difference between the two directions (i.e., resistance of the impedance measured in longitudinal and transverse directions) will have a smaller anisotropy ratio.

## Bioelectrical Impedance Analysis vs. Electrical Impedance Myography

By far, the most cost-effective and easily applied approach currently available for estimating lean body mass and muscle mass is whole-body BIA. One simple way of thinking about EIM is as an improved and enhanced form of BIA. EIM and BIA use the same basic principle of electrical bioimpedance to assess body condition. However, whole-body BIA measures a huge segment of the body at once. Thus, measurements of skeletal muscle, which are of primary interest in studies of aging and sarcopenia, are diluted by many additional tissues, including abdominal fat, bone, gut and bladder contents, and major organs (Androutsos et al., 2015). BIA also is strongly impacted by hydration level since electrical current will always follow the path of least resistance – generally along conductive fluid filled large veins and arteries (O’Brien et al., 2002; Oppliger and Bartok, 2002). Joint replacements can also potentially impact BIA results (Stehaug et al., 2017; Wagner, 2020). Another drawback is that BIA is commonly based on simplistic models in which the torso and limbs are modeled as simple cylinders, thus greatly limiting accuracy and reliability. For instance, agreement between appendicular lean mass assessed by established DXA measures and predicted by BIA is poor [ICC = 0.37 (95%CI: 0.25–0.48)] (Buckinx et al., 2015). BIA is therefore generally considered to have limited sensitivity to muscle mass in sarcopenia (Reiter et al., 2019).

Some researchers have advocated for “segmental BIA” (Ling et al., 2011), in which electrodes are placed in multiple regions or on specific limbs. This likely improves upon the whole-body form of BIA, but segmental BIA is still highly dependent on hydration status as large veins and arteries still remain a factor. In addition, joint position has a major impact, as joint movement can alter electrical current flow in unpredictable ways (Rutkove, 2009). Moreover, segmental BIA can be somewhat ungainly to perform since it involves placement of single electrodes in multiple locations. While EIM has its limitations (discussed in more detail below), it circumvents the major challenges of whole-body and segmental BIA approaches (Table 1). Critically, EIM is relatively unaffected by hydration status (Jia et al., 2014). This is likely because EIM current flow never reaches the low resistance paths of major veins and arteries (Jafarpoor et al., 2013). Also, since current is restricted to muscles, other factors, including gut contents, joint position, and joint replacements do not come into play. Moreover, EIM can provide information on specific muscles or muscle groups and remove added uncertainties of varying inter-electrode distances that impact all other forms of BIA (Sanchez and Rutkove, 2017). Most importantly, EIM is extremely fast and flexible, taking only seconds to perform.

**Table 1:** Whole-body bioelectrical impedance analysis (BIA) vs. segmental BIA vs. electrical muscle impedance (EIM).

Factor	Whole body BIA	Segmental BIA	EIM
Impact of hydration	Very high	High	Negligible
Reliance on models	High	High	None (only raw data used)
Body position	High	Moderate	Negligible
Joint position	Moderate	High	Negligible
Joint replacements	Mild	Moderate, if nearby	Negligible
Gut contents	Moderate	None	None
Abdominal fat	Moderate	None	None
Consistency of interelectrode distances	Slight	Moderate	None (electrode position predetermined)
Muscle specificity	None	Limited	High
Anisotropy assessment	Not possible	Not possible	Readily achievable
Speed of measurement	About 5 min	5 min	20 s per muscle
Cost	Low	Low	Low

While this review focuses almost exclusively on surface EIM methods, it is also possible to perform EIM using needle electrodes placed directly in the muscle (Kwon et al., 2018). This approach has certain benefits, including the ability to measure muscle directly, but given that it is still early in its development, and its invasive nature and potential for sampling error, it is potentially not as relevant to the sarcopenic community. Thus, EIM *via* needle electrode interrogation is not discussed further here.

## Could Electrical Impedance Myography be a Feasible Tool to Evaluate Age-Related Skeletal Muscle Function Deficits?

**Figure 1:**
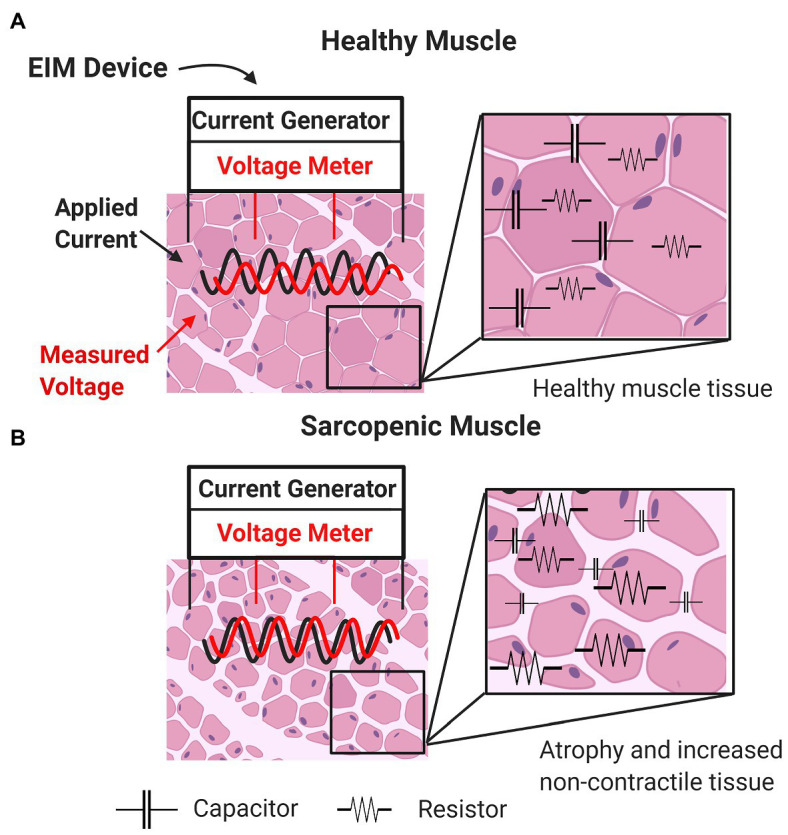
Basic concepts underlying impedance measurements of healthy muscle **(A)** and sarcopenic muscle **(B)** characterized by increased non-contractile tissue (e.g., increased myosteatosis) and smaller myocytes (i.e., atrophied myofibers).

As in all bioimpedance-based technologies, in EIM, a weak, non-detectible (e.g., 400 μA), high frequency (e.g., 1 kHz–1 MHz) electrical current is applied to an area of tissue through two excitation electrodes using multiple input frequencies, and resulting voltages are measured *via* two sense electrodes (Rutkove and Sanchez, 2019). In EIM, electrical current (Figure 1, black sinusoid) is applied *via* two outer surface electrodes, generating a voltage measured by two inner electrodes (Figure 1, red sinusoid). Both intra-rater and inter-rater reliability across sessions have been reported. These data suggest that when care is taken to position the electrodes in the same place, the between-day repeatability for phase angle and resistance is very high in multiple muscles (*r*-values 0.94–0.98; Rutkove et al., 2006; Martinez-Gonzalez et al., 2020). Moreover, alterations in values with a shift in electrode position are fairly modest for small distances, with variations in phase values generally being less than 10% (Rutkove et al., 2005).

**Figure 2:**
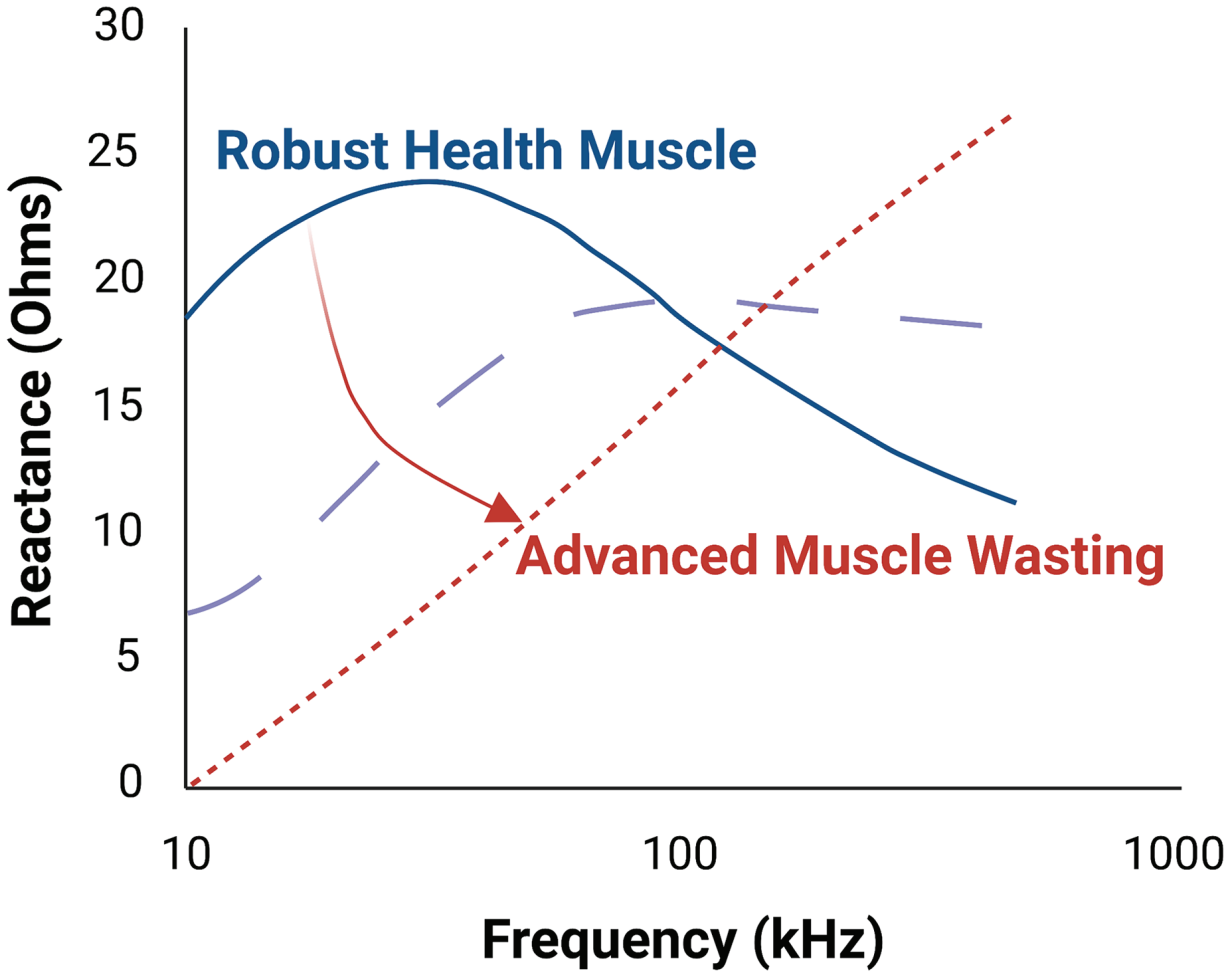
Multifrequency electrical impedance myography (EIM) provides improved tissue characterization in comparison to single frequency EIM. As illustrated here, progressive muscle atrophy yields markedly different tissue reactance, which varies depending on the frequency of the applied electric current. The middle dashed line represents an intermediate state of the muscle.

Changes in sarcopenic muscle are characterized by increased non-contractile tissue (e.g., increased myosteatosis) and smaller myocytes (i.e., atrophied myofibers; Figure 1, bottom panel). Here, the presence of increasing connective tissue, fat, and muscle fiber atrophy alters resistance and reactance. Thus, EIM is sensitive to microscopic morphological changes in tissue, including cell density and size and other tissue features such as fibrosis and myosteatosis. Because there is a strong frequency dependence of EIM signals, performing EIM measurements across a range of frequencies enhances tissue characterization (Figure 2; Table 2; Esper et al., 2006).

**Table 2:** Impedance-compositional relationships in skeletal muscle.

Muscle alteration	Impedance parameter
Myofiber atrophy	Increasing resistance, decreasing reactance values in 30–100 kHz range
Fatty infiltration	Increasing resistance at 300 kHz–1 MHz, decreasing anisotropic ratio
Intracellular abnormalities^1^	Elevations in 1–2 MHz resistance and reactance values

1 e.g., glycogen accumulation and vacuole formation.

While EIM has not yet gained significant attention in the aging field, a significant number of animal (>50) and human (>150) studies on primary and secondary muscle disorders have been published in the extant literature over the past 20 years. This literature base includes studies comparing EIM to muscle histology (Jafarpoor et al., 2013; Arnold et al., 2017; Kapur et al., 2018a,b; Mortreux et al., 2019; Pandeya et al., 2021), theoretical studies assessing current flow (Wang et al., 2011; Pacheck et al., 2016; Kwon et al., 2019), and clinical studies showing EIM’s sensitivity to disease progression and loss of muscle function (Esper et al., 2006; Garmirian et al., 2009; Li et al., 2015; Rutkove et al., 2017; Shefner et al., 2018; Roy et al., 2020). While these investigations are from a relatively small group of investigators and have involved seriously debilitating neuromuscular conditions, they underscore the potential utility of EIM in disorders that impact muscle health, such as age-related skeletal muscle function deficits.

Two recent longitudinal studies, one in adults with amyotrophic lateral sclerosis (ALS; Shefner et al., 2018) and one in boys with Duchene muscular dystrophy (DMD; Rutkove et al., 2017), examined disease progression (rate of change over time) in these conditions and compared EIM to other functional measures. In both studies, EIM was highly sensitive to disease change and muscle function loss and correlated with standard clinical measures of disease progression. Moreover, in the DMD study, initiation of corticosteroids significantly improved EIM values, consistent with corticosteroids’ known clinical benefit (Rutkove et al., 2017).

Moreover, the sensitivity of EIM far surpassed that of standard clinical measures, shown by marked reductions in sample sizes needed for future clinical studies, indicating the utility of EIM as a biomarker for future trials. In the case of DMD, EIM results were similar to those reported previously using MRI (Willcocks et al., 2016). While aging does not wreak such severe injury upon skeletal muscle condition as ALS and DMD, there are many parallels, including myofiber atrophy and fatty infiltration. Importantly, however, EIM is also sensitive to disuse atrophy (Tarulli et al., 2009). For instance, EIM data were obtained from the tibialis anterior muscles of 10 individuals, and the phase at 50 kHz was ~30% lower in immobilized legs when compared to the non-immobilized leg (Tarulli et al., 2009). When normal mobility was restored, the phase of the casted leg increased relative to its initial measurement (Tarulli et al., 2009). This finding is consistent with rodent studies involving spaceflight and hind limb unloading (Li et al., 2013; Sung et al., 2013a; Semple et al., 2020). Thus, EIM could have utility in assessing age-related skeletal muscle deficits. Below, we highlight a series of studies that provide evidence for the utility of EIM in assessing muscle size, composition, and contractile quality in a variety of neuromuscular diseases and conditions.

## Eim is Associated with Myofiber Cross-Sectional Area in Mice

**Figure 3:**
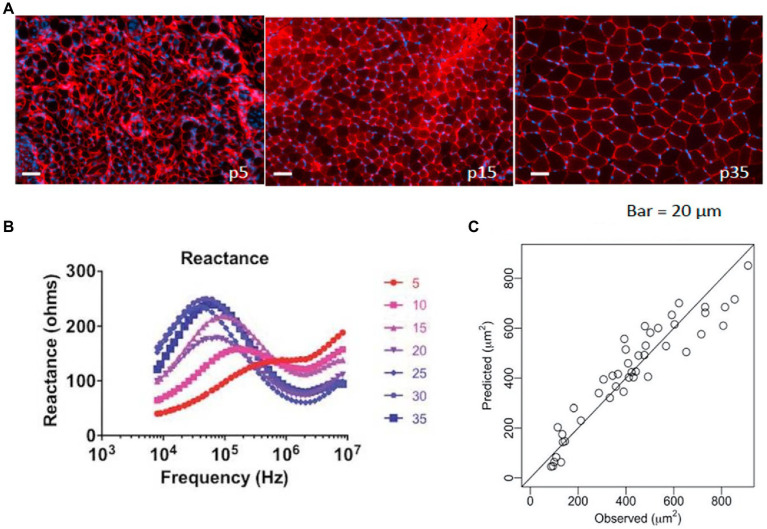
Example of using EIM data to predict muscle fiber size. **(A)** A group of immature mice aged 5–35 days (p15–p35) underwent EIM measurements, were euthanized, and were histologically assessed [note increasing fiber size with increasing age; sections were stained with collagen VI antibodies to identify the cell membranes (red) and 4', 6- diamidino-2-phenylindole (DAPI) to stain nuclei (blue)]. **(B)** Age-related changes are readily observed in the reactance signatures (note the numbers represent the age of the animals in days). **(C)** Using data from all 41 frequencies in a stepwise multiple regression analysis, EIM data predict myofiber size with remarkable accuracy (*r* = 0.95). From Kapur et al. (2018b) Muscle and Nerve, 58: 106–113, 2018. Reprinted with permission by John Wiley Sons.

Relying on the biophysical basis of EIM, prediction modeling approaches could theoretically leverage multifrequency impedance reactance measures to accurately estimate various characteristics of skeletal muscle, including size. Thus, Kapur et al. recently sought to determine whether EIM could be used to approximate myofiber size using standard prediction modeling approaches (Kapur et al., 2018b). Here, wild-type immature mice of varying ages (to provide a range of healthy fiber sizes; Figure 3, upper panel) underwent EIM with a needle electrode array (given their very small size) placed in the gastrocnemius, and myofiber size was subsequently quantified. The multifrequency impedance reactance signature matured along with the animal (Figure 3, bottom left panel). Advanced data analytics, including the least absolute shrinkage and selection operator (Lasso), utilized 240 EIM parameters obtained with each measurement to provide an excellent approximation of mean muscle fiber size across all groups (~90% explained variance; Figure 3, bottom right panel). A similar study was also completed for ALS using a surface electrode array (Kapur et al., 2018b).

### EIM Provides a Means of Quantifying Fat and Connective Tissue Deposition

**Figure 4:**
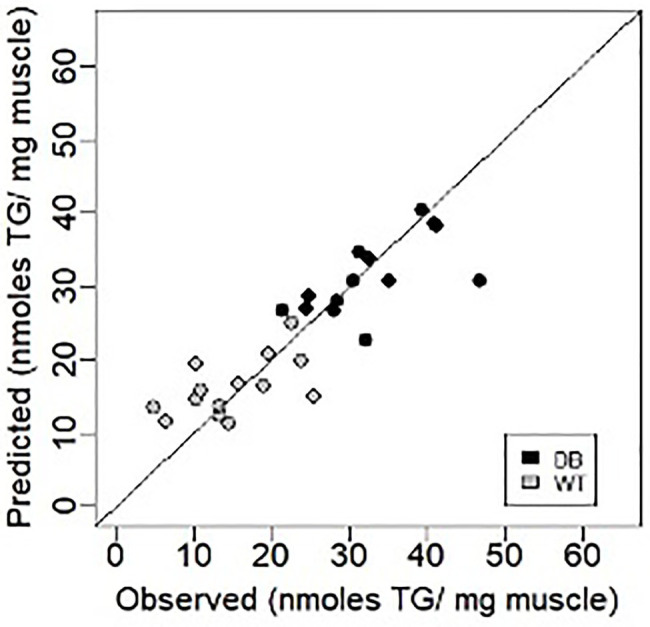
Prediction of muscle triglyceride content using EIM data in the db/db mouse model of obesity (triglyceride; Pandeya et al., 2021). Figure recreated based on data from Pandeya et al. (2021) Muscle and Nerve, 63: 127–140, 2021.

Using a similar Lasso technique to that described above, EIM values were recently shown to tightly correlate with muscle triglyceride content in the db/db mouse model of obesity (Figure 4; Pandeya et al., 2021) and connective tissue content in the mdx muscular dystrophy mouse model (Li et al., 2014). In both cases, raw impedance parameters were significantly associated with actual tissue characteristics (correlation coefficients ranging from 0.65 to 0.85).

### EIM is Associated With Contractility and Contractile Quality

Data obtained from groups of ALS and wild type mice have demonstrated that EIM parameters (e.g., 50 kHz phase and central frequency, the latter being a reactance-based measure derived from multifrequency data) are associated with maximum force production using electrical stimulation of the posterior tibial nerve (Li et al., 2016). EIM measures have also been associated with indices of muscle quality (e.g., echo intensity from ultrasonography, strength normalized to muscle mass) in humans (Longo et al., 2020). The explained variances for these single EIM measures predicting contractile force and indices of muscle quality have ranged 36–77% and will improve as machine learning approaches develop predictive algorithms leveraging numerous EIM measures obtained across multiple frequencies.

### Age-Related Changes in EIM Parameters

**Figure 5:**
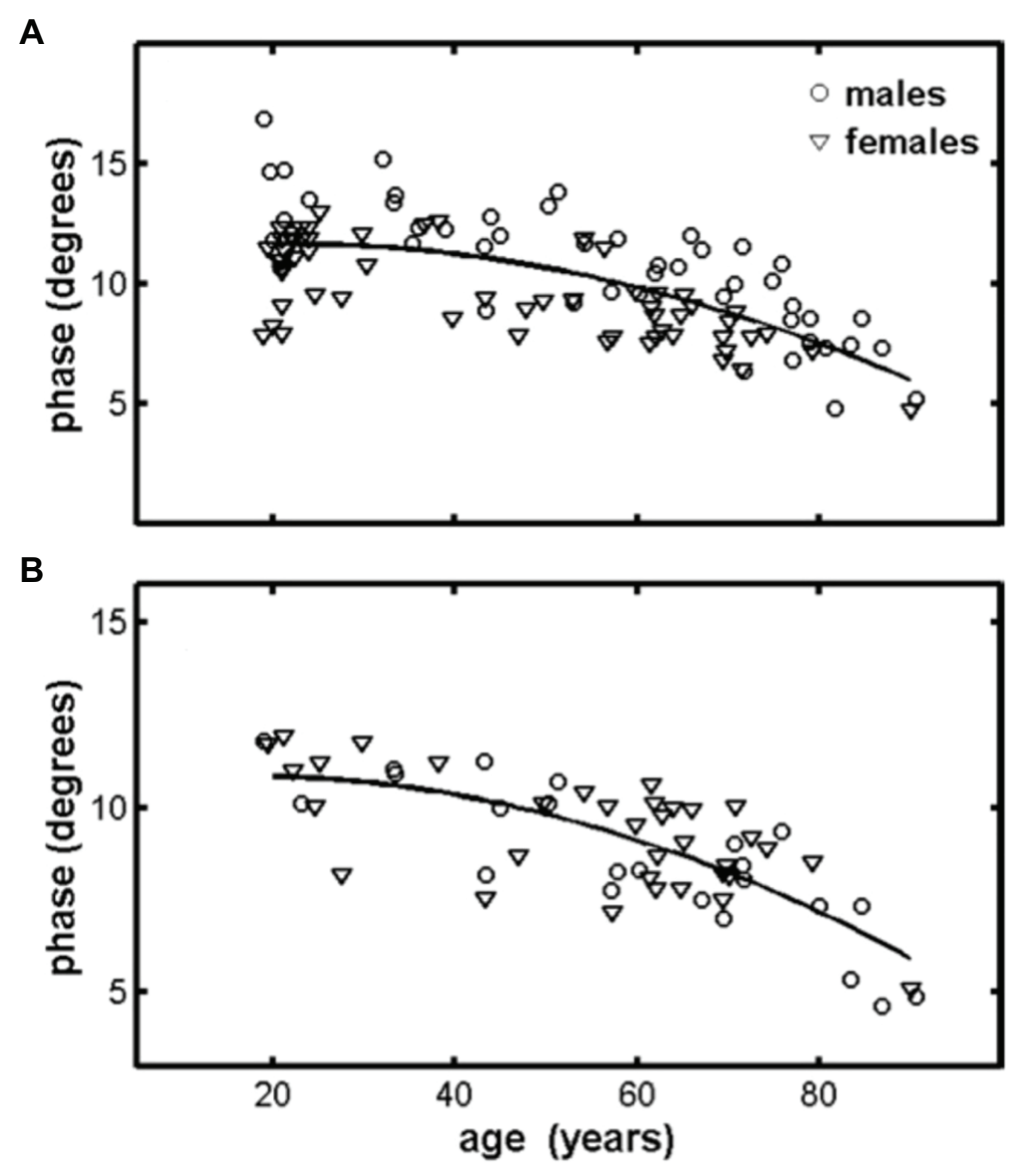
Quadriceps **(A)** and tibialis anterior **(B)** muscles display a reduction in the 50-KHz phase measured using EIM. Lines represent quadratic fits to the combined male–female data for each muscle. Data represent the spatially averaged phase (*θ*_avg_). From Aaron et al. (2006) Physiological Measurement, 953–959. Reprinted with permission by IOP Publishing.

Few studies have examined age-related changes in EIM parameters (Aaron et al., 2006; Kortman et al., 2013; Arnold et al., 2017; Hobson-Webb et al., 2018). One study obtained EIM measurements from the quadriceps and tibialis anterior of 100 healthy adults aged 18–90 years (Aaron et al., 2006). In this study, a quadratic reduction was observed between the EIM 50-kHz phase and increasing age for both muscle groups, with a noted rapid decline starting at ~60 years (Figure 5). This study also longitudinally examined a small group of older adults (*n* = 4) over time (3–5 years), and a notable reduction in phase values was observed in all subjects. In a similar follow-up experiment, Kortman et al. (2013) obtained EIM data (7 upper extremity and 7 lower extremity muscles) from 38 individuals aged 19–50 years and 41 individuals aged 60–85 years. Although the phase values were similar in both groups, reactance and resistance values were ~25% lower in the lower extremities of older individuals than in younger individuals, whereas changes in upper extremity values were not significantly different. This is consistent with prior work suggesting that aging results in more marked degenerative changes in muscle form and function in the lower extremities when compared to the upper extremities (Janssen et al., 2000; Clark and Taylor, 2011).

More recently, Arnold et al. (2017) and Hobson-Webb et al. (2018) examined the potential utility of EIM to assess age-related changes in muscle health. Arnold et al. (2017) compared EIM with other standard measures of muscle structure and function in aged and young mice. EIM demonstrated significantly lower 50-kHz impedance phase and reactance values as well as reduced multifrequency parameters in aged animals. EIM parameters showed good correlation with reduced standard physiological and electrophysiological measures of muscle health in aged mice (e.g., EIM phase and reactance were significantly associated with normalized grip strength, muscle mass, and measures of electrically stimulated contractility). Hobson-Webb et al. (2018) evaluated 27 older adults (mean age: 72 years) and noted exceptionally high intra- and inter-rater reliability for EIM data obtained from the rectus femoris/vastus intermedius muscle complex (*r* = 0.98=0.99). Moreover, they reported significant associations between EIM measures of 50 and 200 kHz resistance and phase values on normalized leg extensor muscle strength and timed up and go test performance. They also observed strong associations between EIM parameters and DXA-derived measures of upper thigh lean mass (e.g., 200-KHz phase *r* = 0.72).

**Figure 6:**
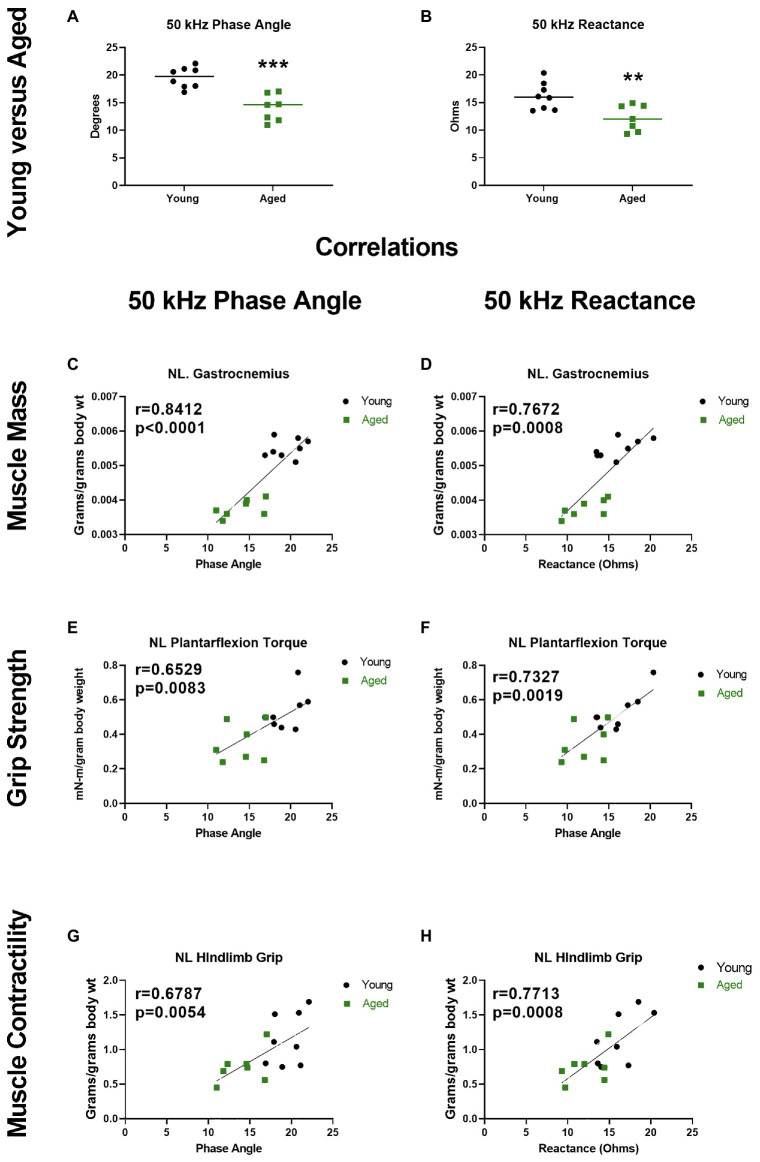
Electrical impedance myography at 50 kHz current frequency detects altered phase angle and reactance in the gastrocnemius muscles of aged rats, and these alterations correlate with muscle status. **(A,B)** Phase angle and reactance were significantly decreased in aged (26 months) vs. young (6 months) F344 rats (unpaired *t* test, *p* = 0.0076 and *p* = 0.0002, respectively). Resistance was not significantly altered in aged rats (data not shown, unpaired *t* test *p* = 0.1207). **(C–H)** Phase and reactance show significant correlations with gastrocnemius wet muscle mass, *in vivo* plantarflexion contractile torque during stimulation at 125 Hz, and hindlimb grip strength (torque and grip normalized to body mass; *r* = Pearson correlation coefficients). ^**^p < 0.01, ^***^p < 0.001. The EIM data presented herein are original data. This data were obtained from the same rats that have been more fully described and characterized for other age-related neuromuscular physiological impairments by Padilla et al. (in press).

While data related to aging and sarcopenia are limited, they suggest that EIM can detect age-related skeletal muscle changes and may represent a convenient and valuable method of quantifying skeletal muscle function deficits in older adults. To further expand on the data supporting the potential utility for EIM to detect age-related skeletal muscle function deficits, in Figure 6, we present original data from young (6 months; *n* = 8) and old (26 months; *n* = 7) F344 rats. These findings demonstrate that both 50 KHz phase and reactance obtained from needle EIM are lower in older vs. younger rats (Figures 6A,B). More importantly, these EIM parameters were associated with measures of muscle mass (Figures 6C,D), muscle strength (Figures 6E,F), and muscle contractility (Figures 6G,H).

## Discussion

### Limitations and Future Directions

In our opinion, there are several limitations of surface EIM that should be noted. The first limitation surrounds contact artifact issues. Contact artifacts can be easily identified and successfully dealt with, usually by application of more saline to the skin, but result in distorted values if disregarded. Another limitation is the impact of the subcutaneous fat layer. Depending on the size of the muscle studied relative to the array, the applied current will flow through skin and subcutaneous fat and impact surface impedance values. There are commercially available units with electrodes designed, based on finite element method modeling (Jafarpoor et al., 2013), to reduce the issue of subcutaneous adiposity, but it is impossible to fully remove the influence of fat. In general, reactance values are less affected than resistance values (Sung et al., 2013b). It remains theoretically possible that the impact of fat could be further reduced by using two sets of electrodes in combination with machine learning approaches. However, it is likely that at a certain level of obesity, surface EIM simply may not work well. Indeed, modeling work has indicated that once subcutaneous fat reaches about 5-cm (~ 2-in) in thickness, very little current will penetrate the muscle using the standard electrode arrangements and inter-electrode distances (Jafarpoor et al., 2013). Still, there is no reason that an electrode array with greater inter-electrode distances could not be developed for such individuals. Similarly, current surface EIM electrode configurations are not able to measure the true anisotropy of muscle tissue well. Nonetheless, because current flow is directionally dependent (i.e., anisotropy; Garmirian et al., 2009), conceptually the degree of anisotropy could also be an indicator of muscle health status in older adults, as myofiber atrophy and increased deposition of isotropic material such as fat and connective tissue will result in decreasing anisotropy. This remains an area for further investigation. Another limitation is that EIM is currently best suited for studying superficial muscles. However, increasing separation of the electrodes would lead to deeper muscle penetration, so it remains theoretically possible to measure deeper muscles. Lastly, the causal factor that EIM aims to measure the volume conductor properties of muscle with the underlying concept being that the health/disease state of muscle can be assessed indirectly by measuring its surface electrical impedance. These volume conduction properties are determined the muscles actual material properties, namely the conductivity and relative permittivity, but these are not directly measured by EIM. Rather, resistance and reactance values are measured, which are not true skeletal muscle properties *per se* (Sanchez et al., 2020). These may be impacted by the subcutaneous fat layer and shape and size of the area being measured. For example, two people may have identical muscle composition and microstructure and identical subcutaneous fat thickness. But because one person had a larger frame and a corresponding greater biceps mass than the other, the EIM resistance and reactance values (but less so the phase) would be somewhat different.

We should explicitly note that we are not suggesting that EIM should be clinically adopted to assess skeletal muscle function deficits in older adults. Clinical adoption, at this time at least, is premature as the there is a lack of convincing human data to warrant the uptake of the method. Moreover, interpretation would be very limited. However, we are advocating for more attention and research be devoted to examining the potential for EIM to be used to assess skeletal muscle health in aging. If this data were supportive of its clinical utility, then it is clear that EIM has notable benefits in that it is very inexpensive, quick, portable, and easy to apply. The most critical next step for development of EIM as a quantitative way to assess skeletal muscle function deficits in older adults is, arguably, translation of current multi-frequency data relating to resistance, reactance, and phase to more easily understand and common metrics of muscle health (e.g., muscle mass, composition). One of the likely reasons EIM has not gained much attention in the sarcopenia field is that these impedance values are not directly reflective of common key outcome variables in clinical studies (e.g., muscle mass and indices of muscle quality). We postulate that EIM measurements could be combined with machine learning approaches to develop prediction equations for these common key outcome variables indicative of muscle health.

### Conclusion

We posed the question of whether EIM could be a feasible tool to evaluate age-related skeletal muscle function deficits. Although we cannot answer this question at this time with certainty, based on the theoretical underpinnings and experimental data discussed above, we believe that EIM has an immense amount to offer – particularly when one considers its speed, cost, ease of use as well as the small size and portability of equipment – to quantifying age-related skeletal muscle deficits. Because EIM provides spectral and anisotropic data on muscle composition, we postulate that deep learning techniques to extract all information buried within the impedance data set could be leveraged to optimize its ability to assess muscle size, contractility, and composition, the critical features of aged and sarcopenic muscle. We are not suggesting that EIM yet be clinically adopted, but we are advocating for more attention and research be devoted to examining the potential for EIM to be used to assess skeletal muscle health in aging.

## Author Contributions

BC, SR, EL, and WA conceived of the manuscript. BC composed it and all other authors reviewed, provided critical input, and revisions, and approved the manuscript. WA, BC, SR, and CP designed the electrical impedance myography experiments, and WA and CP performed the experiments and obtained and analyzed the data.

### Conflict of Interest

BC has received research funding from NMD Pharma, Regeneron Pharmaceuticals, Astellas Pharma Global Development Inc., and RTI Health Solutions for contracted studies involving aging and muscle-related research. In the past 5 years, BC has received consulting fees from Regeneron Pharmaceuticals, Zev Industries, and the Gerson Lehrman Group for consultation specific to age-related muscle weakness. BC is a co-founder with equity of OsteoDx Inc., a company that designs instrumentation to measure the mechanical properties of bone. SR has equity in and serves a consultant and scientific advisor to Myolex Inc., a company that designs impedance devices for clinical and research use; he also is a member of the company’s Board of Directors. The company also has an option to license patented impedance technology of which Rutkove is named as an inventor. EL is employed by and is the President of Myolex Inc. WA has served as a consultant for Genentech, La Hoffmann Roche, Cadent Therapeutics, and Novartis. SR and Neil Lupton also have equity in and serve as consultants and scientific advisors to Haystack Diagnostics Inc., a company that develops needle impedance devices. Both companies have options to license patented impedance technology of which SR is named as an inventor.

## Footnotes

^1^The degree to which a specified material conducts electricity, calculated as the ratio of the current density in the material to the electric field that causes the flow of current. It is the reciprocal of the resistivity.

^2^The ability of a material to store electrical potential energy under the influence of an electric field measured by the ratio of the capacitance of a capacitor with the material as dielectric to its capacitance with vacuum as dielectric.

## References

[ref1] Aaron R. Esper G. J. Shiffman C. A. Bradonjic K. Lee K. S. Rutkove S. B. (2006). Effects of age on muscle as measured by electrical impedance myography. Physiol. Meas. 27, 953–959. 10.1088/0967-3334/27/10/002, PMID: 16951455

[ref2] Aberg P. Nicander I. Holmgren U. Geladi P. Ollmar S. (2003). Assessment of skin lesions and skin cancer using simple electrical impedance indices. Skin Res. Technol. 9, 257–261. 10.1034/j.1600-0846.2003.00017.x, PMID: 12877688

[ref3] Androutsos O. Gerasimidis K. Karanikolou A. Reilly J. J. Edwards C. A. (2015). Impact of eating and drinking on body composition measurements by bioelectrical impedance. J. Hum. Nutr. Diet. 28, 165–171. 10.1111/jhn.12259, PMID: 25158295

[ref4] Arnold W. D. Taylor R. S. Li J. Nagy J. A. Sanchez B. Rutkove S. B. (2017). Electrical impedance myography detects age-related muscle change in mice. PLoS One 12:e0185614. 10.1371/journal.pone.0185614, PMID: 29049394 PMC5648130

[ref5] Bazzocchi A. Filonzi G. Ponti F. Albisinni U. Guglielmi G. Battista G. (2016). Ultrasound: which role in body composition? Eur. J. Radiol. 85, 1469–1480. 10.1016/j.ejrad.2016.04.005, PMID: 27235340

[ref6] Brown B. H. (2003). Electrical impedance tomography (EIT): a review. J. Med. Eng. Technol. 27, 97–108. 10.1080/0309190021000059687, PMID: 12775455

[ref7] Buckinx F. Landi F. Cesari M. Fielding R. A. Visser M. Engelke K.. (2018). Pitfalls in the measurement of muscle mass: a need for a reference standard. J. Cachexia. Sarcopenia Muscle 9, 269–278. 10.1002/jcsm.12268, PMID: 29349935 PMC5879987

[ref8] Buckinx F. Reginster J. Y. Dardenne N. Croisiser J. L. Kaux J. F. Beaudart C.. (2015). Concordance between muscle mass assessed by bioelectrical impedance analysis and by dual energy X-ray absorptiometry: a cross-sectional study. BMC Musculoskelet. Disord. 16:60. 10.1186/s12891-015-0510-9, PMID: 25887598 PMC4369090

[ref9] Carson R. G. (2018). Get a grip: individual variations in grip strength are a marker of brain health. Neurobiol. Aging 71, 189–222. 10.1016/j.neurobiolaging.2018.07.023, PMID: 30172220

[ref10] Chin A. B. Garmirian L. P. Nie R. Rutkove S. B. (2008). Optimizing measurement of the electrical anisotropy of muscle. Muscle Nerve 37, 560–565. 10.1002/mus.20981, PMID: 18404614 PMC2742672

[ref11] Clark B. C. Cook S. B. Ploutz-Snyder L. L. (2007). Reliability of techniques to assess human neuromuscular function in vivo. J. Electromyogr. Kinesiol. 17, 90–101. 10.1016/j.jelekin.2005.11.008, PMID: 16427317

[ref12] Clark B. C. Fernhall B. Ploutz-Snyder L. L. (2006). Adaptations in human neuromuscular function following prolonged unweighting: I. Skeletal muscle contractile properties and applied ischemia efficacy. J. Appl. Physiol. 101, 256–263. 10.1152/japplphysiol.01402.2005, PMID: 16514004

[ref13] Clark B. C. Tavoian D. Goodpaster B. H. Cawthon P. M. Hansen R. D. Manini T. M. (2018). Comment on: "pitfalls in the measurement of muscle mass: a need for a reference standard" by Buckinx et al. J. Cachexia. Sarcopenia Muscle 9, 1269–1271. 10.1002/jcsm.12372, PMID: 30680964 PMC6351671

[ref14] Clark B. C. Taylor J. L. (2011). Age-related changes in motor cortical properties and voluntary activation of skeletal muscle. Curr. Aging Sci. 4, 192–199. 10.2174/1874609811104030192, PMID: 21529329 PMC3184350

[ref15] Correa-de-Araujo R. Addison O. Miljkovic I. Goodpaster B. H. Bergman B. C. Clark R. V.. (2020). Myosteatosis in the context of skeletal muscle function deficit: an interdisciplinary workshop at the national institute on aging. Front. Physiol. 11:963. 10.3389/fphys.2020.00963, PMID: 32903666 PMC7438777

[ref16] Correa-de-Araujo R. Harris-Love M. O. Miljkovic I. Fragala M. S. Anthony B. W. Manini T. M. (2017). The need for standardized assessment of muscle quality in skeletal muscle function deficit and other aging-related muscle dysfunctions: a symposium report. Front. Physiol. 8:87. 10.3389/fphys.2017.00087, PMID: 28261109 PMC5310167

[ref17] Cruz-Jentoft A. J. Baeyens J. P. Bauer J. M. Boirie Y. Cederholm T. Landi F.. (2010). Sarcopenia: European consensus on definition and diagnosis: Report of the European Working Group on Sarcopenia in older people. Age Ageing 39, 412–423. 10.1093/ageing/afq034, PMID: 20392703 PMC2886201

[ref18] Cruz-Jentoft A. J. Bahat G. Bauer J. Boirie Y. Bruyere O. Cederholm T.. (2019). Sarcopenia: revised European consensus on definition and diagnosis. Age Ageing 48, 16–31. 10.1093/ageing/afy169, PMID: 30312372 PMC6322506

[ref19] Delmonico M. J. Kostek M. C. Johns J. Hurley B. F. Conway J. M. (2008). Can dual energy X-ray absorptiometry provide a valid assessment of changes in thigh muscle mass with strength training in older adults? Eur. J. Clin. Nutr. 62, 1372–1378. 10.1038/sj.ejcn.1602880, PMID: 17684523

[ref20] Duchowny K. (2019). Do nationally representative cutpoints for clinical muscle weakness predict mortality? Results from 9 years of follow-up in the health and retirement study. J. Gerontol. A Biol. Sci. Med. Sci. 74, 1070–1075. 10.1093/gerona/gly169, PMID: 30052779 PMC6580687

[ref21] Engelke K. Museyko O. Wang L. Laredo J. D. (2018). Quantitative analysis of skeletal muscle by computed tomography imaging-State of the art. J. Orthop. Translat. 15, 91–103. 10.1016/j.jot.2018.10.004, PMID: 30533385 PMC6260391

[ref22] Engstrom C. M. Loeb G. E. Reid J. G. Forrest W. J. Avruch L. (1991). Morphometry of the human thigh muscles. A comparison between anatomical sections and computer tomographic and magnetic resonance images. J. Anat. 176, 139–156. PMID: 1917669 PMC1260321

[ref23] Epstein B. R. Foster K. R. (1983). Anisotropy in the dielectric properties of skeletal muscle. Med. Biol. Eng. Comput. 21, 51–55. 10.1007/BF02446406, PMID: 6865513

[ref24] Erlandson M. C. Lorbergs A. L. Mathur S. Cheung A. M. (2016). Muscle analysis using pQCT, DXA and MRI. Eur. J. Radiol. 85, 1505–1511. 10.1016/j.ejrad.2016.03.001, PMID: 27005009

[ref25] Esper G. J. Shiffman C. A. Aaron R. Lee K. S. Rutkove S. B. (2006). Assessing neuromuscular disease with multifrequency electrical impedance myography. Muscle Nerve 34, 595–602. 10.1002/mus.20626, PMID: 16881067

[ref26] Gabriel C. Gabriel S. Corthout E. (1996a). The dielectric properties of biological tissues: I. Literature survey. Phys. Med. Biol. 41, 2231–2249. 10.1088/0031-9155/41/11/001, PMID: 8938024

[ref27] Gabriel S. Lau R. W. Gabriel C. (1996b). The dielectric properties of biological tissues: II. Measurements in the frequency range 10 Hz to 20 GHz. Phys. Med. Biol. 41, 2251–2269. 10.1088/0031-9155/41/11/002, PMID: 8938025

[ref28] Gabriel S. Lau R. W. Gabriel C. (1996c). The dielectric properties of biological tissues: III. Parametric models for the dielectric spectrum of tissues. Phys. Med. Biol. 41, 2271–2293. 10.1088/0031-9155/41/11/003, PMID: 8938026

[ref29] Garmirian L. P. Chin A. B. Rutkove S. B. (2009). Discriminating neurogenic from myopathic disease via measurement of muscle anisotropy. Muscle Nerve 39, 16–24. 10.1002/mus.21115, PMID: 19058193 PMC2719295

[ref30] Hansen R. D. Williamson D. A. Finnegan T. P. Lloyd B. D. Grady J. N. Diamond T. H.. (2007). Estimation of thigh muscle cross-sectional area by dual-energy X-ray absorptiometry in frail elderly patients. Am. J. Clin. Nutr. 86, 952–958. 10.1093/ajcn/86.4.952, PMID: 17921370

[ref31] Harris-Love M. O. Avila N. A. Adams B. Zhou J. Seamon B. Ismail C.. (2018). The comparative associations of ultrasound and computed tomography estimates of muscle quality with physical performance and metabolic parameters in older men. J. Clin. Med. 7:340. 10.3390/jcm7100340, PMID: 30308959 PMC6210142

[ref32] Harris-Love M. O. Monfaredi R. Ismail C. Blackman M. R. Cleary K. (2014). Quantitative ultrasound: measurement considerations for the assessment of muscular dystrophy and sarcopenia. Front. Aging Neurosci. 6:172. 10.3389/fnagi.2014.00172, PMID: 25071570 PMC4094839

[ref33] Heymsfield S. B. Adamek M. Gonzalez M. C. Jia G. Thomas D. M. (2014). Assessing skeletal muscle mass: historical overview and state of the art. J. Cachexia. Sarcopenia Muscle 5, 9–18. 10.1007/s13539-014-0130-5, PMID: 24532493 PMC3953319

[ref34] Hind K. Oldroyd B. (2013). In-vivo precision of the GE Lunar iDXA densitometer for the measurement of appendicular and trunk lean and fat mass. Eur. J. Clin. Nutr. 67, 1331–1333. 10.1038/ejcn.2013.210, PMID: 24169463

[ref35] Hobson-Webb L. D. Zwelling P. J. Pifer A. N. Killelea C. M. Faherty M. S. Sell T. C.. (2018). Point of care quantitative assessment of muscle health in older individuals: an investigation of quantitative muscle ultrasound and electrical impedance myography techniques. Geriatrics 3:92. 10.3390/geriatrics3040092, PMID: 31011127 PMC6371147

[ref36] Jafarpoor M. Li J. White J. K. Rutkove S. B. (2013). Optimizing electrode configuration for electrical impedance measurements of muscle via the finite element method. IEEE Trans. Biomed. Eng. 60, 1446–1452. 10.1109/TBME.2012.2237030, PMID: 23314763 PMC3984469

[ref37] Janssen I. Heymsfield S. B. Wang Z. Ross R. (2000). Skeletal muscle mass and distribution in 468 men and women aged 18-88. J. Appl. Physiol. 89, 81–88. 10.1152/jappl.2000.89.1.81, PMID: 10904038

[ref38] Jia L. Sanchez B. Rutkove S. B. (2014). The effect of profound dehydration on electrical impedance of mouseskeletal muscle. Annu. Int. Conf. IEEE Eng. Med. Biol. Soc. 2014, 514–517. 10.1109/EMBC.2014.6943641, PMID: 25570009 PMC4287983

[ref39] Jyvakorpi S. K. Urtamo A. Kivimaki M. Salomaa V. Strandberg T. E. (2020). Association of midlife body composition with old-age health-related quality of life, mortality, and reaching 90 years of age: a 32-year follow-up of a male cohort. Am. J. Clin. Nutr. 112, 1287–1294. 10.1093/ajcn/nqaa230, PMID: 32844221

[ref40] Kapur K. Nagy J. A. Taylor R. S. Sanchez B. Rutkove S. B. (2018a). Estimating myofiber size with electrical impedance myography: a study in amyotrophic lateral sclerosis MICE. Muscle Nerve 58, 713–717. 10.1002/mus.26187, PMID: 30175407 PMC6246784

[ref41] Kapur K. Taylor R. S. Qi K. Nagy J. A. Li J. Sanchez B.. (2018b). “Predicting myofiber size with electrical impedance myography: A study in immature mice.” Muscle Nerve. 10.1002/mus.26111 [Epub ahead of print] PMC6108958 29476692

[ref42] Kim J. Wang Z. Heymsfield S. B. Baumgartner R. N. Gallagher D. (2002). Total-body skeletal muscle mass: estimation by a new dual-energy X-ray absorptiometry method. Am. J. Clin. Nutr. 76, 378–383. 10.1093/ajcn/76.2.378, PMID: 12145010

[ref43] Knapp K. M. Welsman J. R. Hopkins S. J. Shallcross A. Fogelman I. Blake G. M. (2015). Obesity increases precision errors in total body dual-energy x-ray absorptiometry measurements. J. Clin. Densitom. 18, 209–216. 10.1016/j.jocd.2014.06.001, PMID: 25087044

[ref44] Kortman H. G. Wilder S. C. Geisbush T. R. Narayanaswami P. Rutkove S. B. (2013). Age- and gender-associated differences in electrical impedance values of skeletal muscle. Physiol. Meas. 34, 1611–1622. 10.1088/0967-3334/34/12/1611, PMID: 24165434 PMC3895401

[ref45] Kwon H. Di Cristina J. F. Rutkove S. B. Sanchez B. (2018). Recording characteristics of electrical impedance-electromyography needle electrodes. Physiol. Meas. 39:055005. 10.1088/1361-6579/aabb8c, PMID: 29616985 PMC5997185

[ref46] Kwon H. Malik W. Q. Rutkove S. B. Sanchez B. (2019). Separation of subcutaneous fat from muscle in surface electrical impedance myography measurements using model component analysis. IEEE Trans. Biomed. Eng. 66, 354–364. 10.1109/TBME.2018.2839977, PMID: 29993468 PMC6357232

[ref47] Kwon H. Nagy J. A. Taylor R. Rutkove S. B. Sanchez B. (2017). New electrical impedance methods for the in situ measurement of the complex permittivity of anisotropic biological tissues. Phys. Med. Biol. 62, 8616–8633. 10.1088/1361-6560/aa8c95, PMID: 28905814

[ref48] Lee S. Kuk J. L. (2013). Changes in fat and skeletal muscle with exercise training in obese adolescents: comparison of whole-body MRI and dual energy X-ray absorptiometry. Obesity (Silver Spring) 21, 2063–2071. 10.1002/oby.20448, PMID: 23512818 PMC3726562

[ref49] Leong D. P. Teo K. K. Rangarajan S. Lopez-Jaramillo P. Avezum A. Jr. Orlandini A.. (2015). Prognostic value of grip strength: findings from the prospective urban rural epidemiology (PURE) study. Lancet 386, 266–273. 10.1016/S0140-6736(14)62000-6, PMID: 25982160

[ref50] Li J. Geisbush T. R. Rosen G. D. Lachey J. Mulivor A. Rutkove S. B. (2014). Electrical impedance myography for the in vivo and ex vivo assessment of muscular dystrophy (mdx) mouse muscle. Muscle Nerve 49, 829–835. 10.1002/mus.24086, PMID: 24752469 PMC5582805

[ref51] Li J. Jafarpoor M. Bouxsein M. Rutkove S. B. (2015). Distinguishing neuromuscular disorders based on the passive electrical material properties of muscle. Muscle Nerve 51, 49–55. 10.1002/mus.24270, PMID: 24752678 PMC4201890

[ref52] Li J. Spieker A. J. Rosen G. D. Rutkove S. B. (2013). Electrical impedance alterations in the rat hind limb with unloading. J. Musculoskelet. Neuronal Interact. 13, 37–44. PMID: 23445913 PMC3984464

[ref53] Li J. Yim S. Pacheck A. Sanchez B. Rutkove S. B. (2016). Electrical impedance myography to detect the effects of electrical muscle stimulation in wild type and Mdx mice. PLoS One 11:e0151415. 10.1371/journal.pone.0151415, PMID: 26986564 PMC4795734

[ref54] Ling C. H. de Craen A. J. Slagboom P. E. Gunn D. A. Stokkel M. P. Westendorp R. G.. (2011). Accuracy of direct segmental multi-frequency bioimpedance analysis in the assessment of total body and segmental body composition in middle-aged adult population. Clin. Nutr. 30, 610–615. 10.1016/j.clnu.2011.04.001, PMID: 21555168

[ref55] Longo S. Coratella G. Rampichini S. Borrelli M. Scurati R. Limonta E.. (2020). “Local fat content and muscle quality measured by a new electrical impedance myography device: correlations with ultrasound variables.” Eur. J. Sport Sci. 1–12. 10.1080/17461391.2020.1751306 [Epub ahead of print] 32237960

[ref56] Lorne E. Mahjoub Y. Diouf M. Sleghem J. Buchalet C. Guinot P. G.. (2014). Accuracy of impedance cardiography for evaluating trends in cardiac output: a comparison with oesophageal Doppler. Br. J. Anaesth. 113, 596–602. 10.1093/bja/aeu136, PMID: 24871872

[ref57] Maden-Wilkinson T. M. Degens H. Jones D. A. McPhee J. S. (2013). Comparison of MRI and DXA to measure muscle size and age-related atrophy in thigh muscles. J. Musculoskelet. Neuronal Interact. 13, 320–328. PMID: 23989253

[ref58] Martinez-Gonzalez M. Montilla-Herrador J. Garcia-Vidal J. A. Escolar-Reina P. Gacto-Sanchez M. Medina-Mirapeix F. (2020). Intra- and inter-rater reliability of electrical impedance myography using adhesive electrodes in healthy volunteers. J. Electromyogr. Kinesiol. 55:102456. 10.1016/j.jelekin.2020.102456, PMID: 32905888

[ref59] McGrathR.ErlandsonK. M.VincentB. M.HackneyK. J.HerrmannS. D.ClarkB. C. (2019a). Decreased handgrip strength is associated with impairments in each autonomous living task for aging adults in the United States. J. Frailty Aging 8, 141–145. 10.14283/jfa.2018.47, PMID: 31237315

[ref60] McGrath R. Vincent B. M. Peterson M. D. Jurivich D. A. Dahl L. J. Hackney K. J.. (2019b). Weakness may have a causal association with early mortality in older americans: a matched cohort analysis. J. Am. Med. Dir. Assoc. 21, 621–626.e2. 10.1016/j.jamda.2019.10.016, PMID: 31786197 PMC7186143

[ref61] Morse C. I. Degens H. Jones D. A. (2007). The validity of estimating quadriceps volume from single MRI cross-sections in young men. Eur. J. Appl. Physiol. 100, 267–274. 10.1007/s00421-007-0429-4, PMID: 17342544

[ref62] Mortreux M. Semple C. Riveros D. Nagy J. A. Rutkove S. B. (2019). Electrical impedance myography for the detection of muscle inflammation induced by lambda-carrageenan. PLoS One 14:e0223265. 10.1371/journal.pone.0223265, PMID: 31574117 PMC6773213

[ref63] Nelson M. E. Fiatarone M. A. Layne J. E. Trice I. Economos C. D. Fielding R. A.. (1996). Analysis of body-composition techniques and models for detecting change in soft tissue with strength training. Am. J. Clin. Nutr. 63, 678–686. 10.1093/ajcn/63.5.678, PMID: 8615349

[ref64] Nijholt W. Scafoglieri A. Jager-Wittenaar H. Hobbelen J. S. M. van der Schans C. P. (2017). The reliability and validity of ultrasound to quantify muscles in older adults: a systematic review. J. Cachexia. Sarcopenia Muscle 8, 702–712. 10.1002/jcsm.12210, PMID: 28703496 PMC5659048

[ref65] O’Brien C. Young A. J. Sawka M. N. (2002). Bioelectrical impedance to estimate changes in hydration status. Int. J. Sports Med. 23, 361–366. 10.1055/s-2002-33145, PMID: 12165888

[ref66] Oppliger R. A. Bartok C. (2002). Hydration testing of athletes. Sports Med. 32, 959–971. 10.2165/00007256-200232150-00001, PMID: 12457417

[ref67] Pacheck A. Mijailovic A. Yim S. Li J. Green J. R. McIlduff C. E.. (2016). Tongue electrical impedance in amyotrophic lateral sclerosis modeled using the finite element method. Clin. Neurophysiol. 127, 1886–1890. 10.1016/j.clinph.2015.11.046, PMID: 26750579 PMC4828196

[ref68] Padilla C. J. Harrigan M. E. Schwab J. M. Rutkove S. B. Rich M. M. Clark B. C.. (in press). Profiling age-related muscle weakness and wasting: neuromuscular junction transmission as a driver of age-related physical decline. Geroscience. 10.1007/s11357-021-00369-3 PMC8190265 33895959

[ref69] Pandeya S. R. Nagy J. A. Riveros D. Semple C. Taylor R. S. Mortreux M.. (2021). Predicting myofiber cross-sectional area and triglyceride content with electrical impedance myography: a study in db/db mice. Muscle Nerve 63, 127–140. 10.1002/mus.27095, PMID: 33063867 PMC8891989

[ref70] Phillips R. (2013). Prostate cancer: increasing the potential of prostate biopsies with bioimpedance spectroscopy. Nat. Rev. Urol. 10:558. 10.1038/nrurol.2013.215, PMID: 24042562

[ref71] Rantanen T. (2003). Muscle strength, disability and mortality. Scand. J. Med. Sci. Sports 13, 3–8. 10.1034/j.1600-0838.2003.00298.x, PMID: 12535311

[ref72] Rantanen T. Avlund K. Suominen H. Schroll M. Frandin K. Pertti E. (2002). Muscle strength as a predictor of onset of ADL dependence in people aged 75 years. Aging Clin. Exp. Res. 14, 10–15. PMID: 12475129

[ref73] Rantanen T. Guralnik J. M. Foley D. Masaki K. Leveille S. Curb J. D.. (1999). Midlife hand grip strength as a predictor of old age disability. JAMA 281, 558–560. 10.1001/jama.281.6.558, PMID: 10022113

[ref74] Rantanen T. Guralnik J. M. Izmirlian G. Williamson J. D. Simonsick E. M. Ferrucci L.. (1998). Association of muscle strength with maximum walking speed in disabled older women. Am. J. Phys. Med. Rehabil. 77, 299–305. 10.1097/00002060-199807000-00008, PMID: 9715919

[ref75] Rantanen T. Harris T. Leveille S. G. Visser M. Foley D. Masaki K.. (2000). Muscle strength and body mass index as long-term predictors of mortality in initially healthy men. J. Gerontol. A Biol. Sci. Med. Sci. 55, M168–M173. 10.1093/gerona/55.3.M168, PMID: 10795731

[ref76] Reimers K. Reimers C. D. Wagner S. Paetzke I. Pongratz D. E. (1993). Skeletal muscle sonography: a correlative study of echogenicity and morphology. J. Ultrasound Med. 12, 73–77. 10.7863/jum.1993.12.2.73, PMID: 8468739

[ref77] Reiter R. Iglseder B. Treschnitzer W. Alzner R. Mayr-Pirker B. Kreutzer M.. (2019). Quantifying appendicular muscle mass in geriatric inpatients: performance of different single frequency BIA equations in comparison to dual X-ray absorptiometry. Arch. Gerontol. Geriatr. 80, 98–103. 10.1016/j.archger.2018.10.010, PMID: 30419483

[ref78] Roy B. Rutkove S. B. Nowak R. J. (2020). Electrical impedance myography as a biomarker of inclusion body myositis: a cross-sectional study. Clin. Neurophysiol. 131, 368–371. 10.1016/j.clinph.2019.10.030, PMID: 31865137

[ref79] Rozenberg D. Martelli V. Vieira L. Orchanian-Cheff A. Keshwani N. Singer L. G.. (2017). Utilization of non-invasive imaging tools for assessment of peripheral skeletal muscle size and composition in chronic lung disease: a systematic review. Respir. Med. 131, 125–134. 10.1016/j.rmed.2017.08.007, PMID: 28947019

[ref80] Rutkove S. B. (2009). Electrical impedance myography: background, current state, and future directions. Muscle Nerve 40, 936–946. 10.1002/mus.21362, PMID: 19768754 PMC2824130

[ref81] Rutkove S. B. Kapur K. Zaidman C. M. Wu J. S. Pasternak A. Madabusi L.. (2017). Electrical impedance myography for assessment of Duchenne muscular dystrophy. Ann. Neurol. 81, 622–632. 10.1002/ana.24874, PMID: 28076894 PMC5444980

[ref82] Rutkove S. B. Lee K. S. Shiffman C. A. Aaron R. (2006). Test-retest reproducibility of 50 kHz linear-electrical impedance myography. Clin. Neurophysiol. 117, 1244–1248. 10.1016/j.clinph.2005.12.029, PMID: 16644269

[ref83] Rutkove S. B. Partida R. A. Esper G. J. Aaron R. Shiffman C. A. (2005). Electrode position and size in electrical impedance myography. Clin. Neurophysiol. 116, 290–299. 10.1016/j.clinph.2004.09.002, PMID: 15661107

[ref84] Rutkove S. B. Sanchez B. (2019). Electrical impedance methods in neuromuscular assessment: an overview. Cold Spring Harb. Perspect. Med. 9:a034405. 10.1101/cshperspect.a034405, PMID: 30291145 PMC6771359

[ref85] Sanchez B. Martinsen O. G. Freeborn T. J. Furse C. M. (2020). Electrical impedance myography: a critical review and outlook. Clin. Neurophysiol. 132, 338–344. 10.1016/j.clinph.2020.11.014, PMID: 33450556 PMC7880888

[ref86] Sanchez B. Rutkove S. B. (2017). Electrical impedance myography and its applications in neuromuscular disorders. Neurotherapeutics 14, 107–118. 10.1007/s13311-016-0491-x, PMID: 27812921 PMC5233633

[ref87] Sasaki H. Kasagi F. Yamada M. Fujita S. (2007). Grip strength predicts cause-specific mortality in middle-aged and elderly persons. Am. J. Med. 120, 337–342. 10.1016/j.amjmed.2006.04.018, PMID: 17398228

[ref88] Seeman T. E. Merkin S. S. Crimmins E. M. Karlamangla A. S. (2010). Disability trends among older Americans: national health and nutrition examination surveys, 1988-1994 and 1999-2004. Am. J. Public Health 100, 100–107. 10.2105/AJPH.2008.157388, PMID: 19910350 PMC2791257

[ref89] Semple C. Riveros D. Sung D. M. Nagy J. A. Rutkove S. B. Mortreux M. (2020). Using electrical impedance myography as a biomarker of muscle deconditioning in rats exposed to micro- and partial-gravity analogs. Front. Physiol. 11:557796. 10.3389/fphys.2020.557796, PMID: 33041858 PMC7522465

[ref90] Shefner J. M. Rutkove S. B. Caress J. B. Benatar M. David W. S. Cartwright M. S.. (2018). Reducing sample size requirements for future ALS clinical trials with a dedicated electrical impedance myography system. Amyotroph. Lateral Scler. Frontotemporal Degener. 19, 555–561. 10.1080/21678421.2018.1510008, PMID: 30265154 PMC6438779

[ref91] Sipila S. Suominen H. (1993). Muscle ultrasonography and computed tomography in elderly trained and untrained women. Muscle Nerve 16, 294–300. 10.1002/mus.880160309, PMID: 8446128

[ref92] Stehaug O. M. Bogen B. Kristoffersen M. H. Ranhoff A. H. (2017). Bones, blood and steel: how bioelectrical impedance analysis is affected by hip fractures and surgical implants. J. Electr. Bioimpedance 8, 54–59. 10.5617/jeb.4104

[ref93] Sung M. Li J. Spieker A. J. Spatz J. Ellman R. Ferguson V. L.. (2013a). Spaceflight and hind limb unloading induce similar changes in electrical impedance characteristics of mouse gastrocnemius muscle. J. Musculoskelet. Neuronal Interact. 13, 405–411. PMID: 24292610 PMC4653813

[ref94] Sung M. Spieker A. J. Narayanaswami P. Rutkove S. B. (2013b). The effect of subcutaneous fat on electrical impedance myography when using a handheld electrode array: the case for measuring reactance. Clin. Neurophysiol. 124, 400–404. 10.1016/j.clinph.2012.07.013, PMID: 22917581 PMC3543755

[ref95] Syddall H. Cooper C. Martin F. Briggs R. Aihie Sayer A. (2003). Is grip strength a useful single marker of frailty? Age Ageing 32, 650–656. 10.1093/ageing/afg111, PMID: 14600007

[ref96] Tarulli A. W. Duggal N. Esper G. J. Garmirian L. P. Fogerson P. M. Lin C. H.. (2009). Electrical impedance myography in the assessment of disuse atrophy. Arch. Phys. Med. Rehabil. 90, 1806–1810. 10.1016/j.apmr.2009.04.007, PMID: 19801075 PMC2829834

[ref97] Tavoian D. Ampomah K. Amano S. Law T. D. Clark B. C. (2019). Changes in DXA-derived lean mass and MRI-derived cross-sectional area of the thigh are modestly associated. Sci. Rep. 9:10028. 10.1038/s41598-019-46428-w, PMID: 31296891 PMC6624257

[ref98] Toso S. Piccoli A. Gusella M. Menon D. Bononi A. Crepaldi G.. (2000). Altered tissue electric properties in lung cancer patients as detected by bioelectric impedance vector analysis. Nutrition 16, 120–124. 10.1016/S0899-9007(99)00230-0, PMID: 10696635

[ref99] U.N. (2017). United Nations World Population Aging 2017 P.D. Department of Economic and Social Affairs. New York, United Nations.

[ref100] Visser M. Goodpaster B. H. Kritchevsky S. B. Newman A. B. Nevitt M. Rubin S. M.. (2005). Muscle mass, muscle strength, and muscle fat infiltration as predictors of incident mobility limitations in well-functioning older persons. J. Gerontol. A Biol. Sci. Med. Sci. 60, 324–333. 10.1093/gerona/60.3.324, PMID: 15860469

[ref101] Wagner D. R. (2020). Case study: effect of surgical metal implant on single frequency bioelectrical impedance measures of an athlete. Phys. Rep. 8:e14464. 10.14814/phy2.14464, PMID: 32472735 PMC7260760

[ref102] Wang L. L. Ahad M. McEwan A. Li J. Jafarpoor M. Rutkove S. B. (2011). Assessment of alterations in the electrical impedance of muscle after experimental nerve injury via finite-element analysis. IEEE Trans. Biomed. Eng. 58, 1585–1591. 10.1109/TBME.2011.2104957, PMID: 21224171

[ref103] Warzecha M. Amarowicz J. Berwecka M. Czerwinski E. Kumorek A. (2020). Relation between risk of falls, sarcopenia and parameters assessing quality of skeletal muscles in a group of postmenopausal women. Prz Menopauzalny 19, 123–129. 10.5114/pm.2020.99617, PMID: 33100947 PMC7573338

[ref104] Willcocks R. J. Rooney W. D. Triplett W. T. Forbes S. C. Lott D. J. Senesac C. R.. (2016). Multicenter prospective longitudinal study of magnetic resonance biomarkers in a large duchenne muscular dystrophy cohort. Ann. Neurol. 79, 535–547. 10.1002/ana.24599, PMID: 26891991 PMC4955760

